# Prison categorization policy in the United Kingdom

**DOI:** 10.3389/fsoc.2025.1465599

**Published:** 2025-02-19

**Authors:** Scott Thomas, Jonathan Glazzard

**Affiliations:** ^1^Independent Researcher, Leicester, United Kingdom; ^2^Faculty of Arts, Cultures and Education, School of Education, University of Hull, Hull, United Kingdom

**Keywords:** prison, category, prisoner, mental health, policy

## 1 Brief literature review

In the United Kingdom (UK) and elsewhere, different types of prisons serve different purposes. These range from high-security or “closed” prisons to low-security or “open” prisons, where prisoners have considerably more freedom. In the UK, the type of prison which is allocated to a prisoner is dependent upon the category that has been assigned to them, following an assessment of risk. Several sociological studies have identified the detrimental impacts of incarceration on prisoners, particularly the impacts associated with loss of liberty (Crewe, 2009; Mathiesen, 1965; Ugelvik, 2014). However, these studies were conducted in high security prisons, where the deprivation of liberty is most acute.

More recently, a range of sociological studies have explored prisoners' experiences of open prisons (Abrahamsen, 2017; Lundeberg et al., 2018; Maier, 2020; Mjåland and Laursen, 2021; Neumann, 2012; Nielsen, 2012; Pakes, 2020; Pettersson, 2017; Shammas, 2014, 2015a,b; Statham et al., 2020). Although some literature highlights positive staff/prisoner relationships (Pakes, 2020), more humane environments (Pettersson, 2017) and the positive role of open prisons in rehabilitating prisoners (Lundeberg et al., 2018), other literature has emphasized the “pains of freedom” (Shammas, 2014) that are experienced during periods of incarceration in open prisons, including prisoners being in a permanent state of “pre-release” (Shammas, 2014), and the increased responsibility placed on prisoners to prove that they can be trusted. Research also demonstrates that prisoners in open prisons may erect their own “inner bars” to remind themselves that they must remain complaint and governable (Maier, 2020; Neumann, 2012).

Open prisons were established in England, Wales and the United States in the 20th century (Mjåland et al., 2023). England's first open prison, New Hall Camp, was opened in 1934 in Wakefield. However, following a series of security lapses in the 1960s, including some high-profile escapes, the Mountbatten Report (Klare, 1968) suggested a new type of prisoner classification (A, B, C, D), with category D being reserved for open prisons. Price (2000) argued that this classification system is the “most important internal procedure” in the Prison Service, overruling all other policies.

Open prisons serve a resettlement function and cater for prisoners on longer sentences who, following serving time in high-security prisons, can serve the final part of their sentences in less restricted environments as a preparation for release into the community (Mjåland et al., 2023). The move to a less restrictive prison environment supports a more gradual progression toward freedom, thus facilitating the reintegration of prisoners into the community upon release (Ministry of Justice, 2020). Overall, evidence shows that open prisons are experienced as safer, less restrictive and less degrading institutions than closed prisons (Mjåland et al., 2023), and therefore it is unsurprising that prisoners may seek to be assigned category D status. Thus, “Distinguishing between closed and open prisons therefore provides an avenue to explore *how much* liberty is taken away from captives by the state, and with what effects” (Mjåland et al., 2023, p. 1657).

## 2 Critical commentary on prison categorization policy

This opinion piece draws on the lived experiences of a life sentenced prisoner to highlight the difficulties associated with current policy on prisoner categorization in the UK. The recommendations at the end of this article are designed to offer solutions that will allow for a better and more streamlined approach to prisoner security categorization. These recommendations will enable limited resources to be directed at risk management and rehabilitation.

The *Security Categorization Policy Framework* (HM Prison and Probation Service, 2024) suggests that most prisoners should be categorized as C status. This has resulted in a larger number of prisons holding category C prisoners. The definition of the 4 security categories of adult male prisoners is:

- Category A: Those whose escape would be highly dangerous to the public, the police or the security of the State, and for whom the aim must be to make escape impossible.
- Category B: Offenders whose assessed risks require that they are held in the closed estate and who need security measures additional to those in a standard closed prison.
- Category C: Offenders who are assessed as requiring standard closed conditions and do not need additional security.
- Category D: Offenders who are either assessed as presenting a low risk or whose previously identified risk factors are now assessed as manageable in low security conditions.

To be eligible for consideration for category D open prison conditions, prisoners must be at the prescribed point in their sentence. In addition, the prisoner must be assessed as being low risk in relation to absconding, low risk to the public or have a suitable plan in place to manage identified risk, being unlikely to continue criminality in custody and also unlikely to take advantage of the low security and disrupt the good order or regime of the open prison estate.

Security categorization is a risk management process, the purpose of which is to ensure that those sentenced to custody are assigned the appropriate security category in relation to their risk of escape, the risk of harm to the public, the potential for ongoing criminality in custody, and the risk that they will exhibit violent or controlling behavior that adversely impacts on the safety of others and the good order of the prison.

Within the category C estate, many prisoners are short-term prisoners who are seeking to be re-categorized to open prison category D status. Open prisons have minimal perimeter and physical security features and are used for those who are specifically assessed as suitable for conditions of low security. Prisoners are aware of the date that they are eligible for a review for category D status, but within the category C estate the number of prisoners being processed, and under review at any one time, is currently overwhelming the limited resources of the Offender Management Unit.

The focus of prisoners from the early days following sentence until release are the Security Categorization reviews. The *Security Categorization Policy Framework* (HM Prison and Probation Service, 2024) is only 22 pages long but has a significant impact on staffing resources and is costly to implement.

When a prisoner first arrives in prison as a convicted person, they go through a process of initial categorization. This is a risk assessment process which arguably is extremely subjective. Many disagreements between staff, including managers, about the initial categorization decision take place. There are different schools of thought in relation to this. Some staff tend to assign anyone with a sentence of 10 years, or over, to category B, without consideration of other factors. This was in fact the official policy several years ago when a simple flow chart was used to determine a person's initial category, with length of sentence, and the type of offence being the two prominent factors in determining the appropriate categorization. In more recent years, some staff tend to assign category C status, unless there is clear evidence to suggest the need for higher security conditions.

The focus for prisoners is the desire to achieve a lower security category status. Some prisoners refer to this as “chasing the cat”. Prisoners are consumed with trying to get a category review to achieve a lower categorization. This can hinder positive rehabilitation and risk reduction. Prisoners “play the game” and say what is needed simply to try to obtain a positive outcome at their category review. Given that the reviews require processing, the workload and time implications for prison staff are significant. More effective ways of managing progression through sentences are required to increase efficiency.

## 3 Recommendations

We suggest two recommendations which are outlined below.

- Implement a commissioned study:

A study should be commissioned to explore the idea of removing the current categories of A, B, C and D, and adopting the following new classifications:

Category A: Reserved for those assessed as very high risk. We suggest keeping the title of category “A” as this is a well-established category reserved for the most serious offenders. This category comes with its own set of rules and regulations that govern how those categorized as “A” will be managed.

Closed category: For those not suitable for open conditions.

Open category: For those assessed as low risk and/or who can be trusted in an open prison. Open prisons should be for those who can identify a benefit to the added benefits of being held in an open prison.

A new model of A, closed, open categorisations will reduce workload within offender management units and allow more focus on risk management and risk reduction. This model is already in use within the female prison estate and appears to work well. The immediate impact that will be seen is the stability of the population, in terms of prisoner transfers. The cost implications will be significant due to the fact that prisoner transport and the administration involved in transferring prisoners between prisons will be greatly reduced. The medium to long term impact will be the ability of the prison service to work more closely with offenders in a structured and uninterrupted way. This will see better and more tailored interventions that will help to manage and reduce risk of recidivism.

- Develop an information campaign:

A comprehensive information campaign should be developed to help inform prisoners and prison staff of the correct processes that are currently in place for managing prisoners' security category reviews. A well-considered and strategically deployed information campaign will serve to manage expectations, which, for prisoners, will help reduce stress and anxiety. Communication is very poor in prisons and is a cause of self-harm, anger and violence.
